# Apremilast for the treatment of generalized granuloma annulare: A case series of 8 patients

**DOI:** 10.1016/j.jdcr.2023.06.011

**Published:** 2023-06-17

**Authors:** Tejas P. Joshi, Jaime Tschen

**Affiliations:** a School of Medicine, Baylor College of Medicine, Houston, Texas; b St. Joseph Dermatopathology, Houston, Texas

**Keywords:** apremilast, disseminated granuloma annulare, generalized granuloma annulare, granuloma annulare, phosphodiesterase 4 inhibition

*To the Editor:* We read with interest the case series by Blum and Altman^1^ exploring the role of apremilast in the treatment of generalized granuloma annulare (GA). GA is a challenging entity to treat, and despite its significant psychosocial impact, there exists a paucity of treatment options for this condition.^2^^,^^3^ Here, we present a case series of 8 patients with generalized GA treated with apremilast.

Eight patients with generalized GA (6 women and 2 men) ranging in age from 52 to 80 years (median, 64 years) were treated with apremilast 30 mg twice daily. A variable response to apremilast treatment was observed (Table I). Three patients experienced complete clearance of GA: patient 2 had complete lesion clearance at 6 months, with durable remission at 2-year follow-up; patient 4 had 90% lesion clearance at 6 months, at which point she discontinued apremilast and has sustained remission at 1 year; and patient 7 also experienced a complete response to treatment at 6 months with sustained response at 1 year. However, 3 patients failed apremilast—patient 1 had decreased lesion erythema at 3 months, but the erythema returned at 6 months of treatment; patient 3 had no response at 6 months; and patient 6 had decreased lesion erythema at 5 months, but there was no change in lesion size. Patient 8 had complete clearance of lesions at 4 months, but upon down-titration of the apremilast dose to 30 mg once daily, the patient began to experience flares. She then discontinued apremilast and has persistent lesions at 1 year follow-up. Patient 5 experienced a partial response at 6 months, but was lost to follow-up. Apremilast was generally well tolerated, except in 1 patient, who reported diarrhea.

**Table I:** Patients with generalized granuloma annulare treated with apremilast 30 mg twice daily and their clinical response

Pt	Age, sex	GA duration prior to treatment	Clinical response	Side effects
1	69, F	3 mo	Decreased erythema at 3 mo with relapse at 6 mo	None
2	80, F	1 mo	Complete lesion clearance at 6 mo, durable remission at 2 y	None
3	60, F	1 y	No response at 6 mo	None
4	62, M	6 mo	90% clearance at 6 mo, with continued improvement after discontinuing apremilast	None
5	67, F	2 y	Partial response at 6 mo	None
6	66, M	3 mo	Decreased erythema at 5 mo, but no change in lesion number	Diarrhea, led to discontinuation
7	56, F	6 y	Complete clearance at 3 mo with sustained response at 1 y	None
8	52, F	1 y	Near complete response at 4 mo with flares after down-titrating dose to 30 mg once daily; persistent lesions at 1 y after discontinuing treatment	None

*F*, Female; *GA*, granuloma annulare; *M*, male; *Pt*, patient.

Currently, there are 9 other cases of generalized GA treated with apremilast in the literature; all patients in these case reports responded to treatment.^1^^,^^4^, ^5^, ^6^ However, these case studies are limited by publication bias and likely overestimate the true therapeutic potential of apremilast in treating generalized GA. To our knowledge, our case series of 8 patients is the largest to date that shows apremilast to be a potential therapy in some, but not all, generalized GA patients.

As Blum and Altman^1^ point out, apremilast, which downregulates tumor necrosis factor-α and interferon gamma and through phosphodiesterase-4 inhibition, seems a mechanistically reasonable therapy for GA because GA also displays T-helper 1 cytokine dysregulation. However, recent research suggests that T-helper 2 cell dysregulation may also be a component of GA pathogenesis.^7^^,^^8^ It is possible that a differing balance of the 2 competing cytokine pathways among different patients results in a heterogenous response to treatment.

Our case series is limited by the lack of standardized outcome measures for GA. Nonetheless, our case series illustrates that apremilast may be considered as a treatment option in some patients with generalized GA, although more than half the patients may experience a suboptimal response. More upstream therapies (eg, Janus kinase inhibitors) may be explored in patients who fail apremilast or biologics.^9^

## Conflicts of interest

None disclosed.
